# Development of anti-Crimean-Congo hemorrhagic fever virus Gc and NP-specific ELISA for detection of antibodies in domestic animal sera

**DOI:** 10.3389/fvets.2022.913046

**Published:** 2022-08-25

**Authors:** Sandra Belij-Rammerstorfer, Georgina Limon, Emmanuel A. Maze, Kayleigh Hannant, Ellen Hughes, Simona R. Tchakarova, Tsviatko Alexandrov, Blandina T. Mmbaga, Brian Willett, George Booth, Nicholas A. Lyons, Natalie Baker, Kelly M. Thomas, Daniel Wright, Jack Saunders, Clare Browning, Ginette Wilsden, Miles Carroll, Roger Hewson, Bryan Charleston, Teresa Lambe, Anna B. Ludi

**Affiliations:** ^1^Nuffield Department of Medicine, The Jenner Institute, University of Oxford, Oxford, United Kingdom; ^2^The Pirbright Institute, Woking, United Kingdom; ^3^Medical Research Council (MRC)-University of Glasgow Centre for Virus Research (CRV), Glasgow, United Kingdom; ^4^Bulgarian Food Safety Agency, Sofia, Bulgaria; ^5^Kilimanjaro Clinical Research Institute (KCRI), Kilimanjaro Christian Medical Centre, Moshi, Tanzania; ^6^Kilimanjaro Christian Medical University College, Moshi, Tanzania; ^7^Research and Evaluation, UK Health Security Agency, Porton Down, United Kingdom; ^8^Pandemic Science Institute, Wellcome Centre for Human Genetics, Nuffield Department of Medicine, Oxford University, Oxford, United Kingdom; ^9^Department of Infection Biology, London School of Hygiene and Tropical Medicine, London, United Kingdom

**Keywords:** Crimean-Congo hemorrhagic fever, ELISA, domestic animals, antibody response, CCHFV Gc, CCHFV NP

## Abstract

Crimean-Congo hemorrhagic fever (CCHF) is a priority emerging disease. CCHF, caused by the CCHF virus (CCHFV), can lead to hemorrhagic fever in humans with severe cases often having fatal outcomes. CCHFV is maintained within a tick-vertebrate-tick cycle, which includes domestic animals. Domestic animals infected with CCHFV do not show clinical signs of the disease and the presence of antibodies in the serum can provide evidence of their exposure to the virus. Current serological tests are specific to either one CCHFV antigen or the whole virus antigen. Here, we present the development of two in-house ELISAs for the detection of serum IgG that is specific for two different CCHFV antigens: glycoprotein Gc (CCHFV Gc) and nucleoprotein (CCHFV NP). We demonstrate that these two assays were able to detect anti-CCHFV Gc-specific and anti-CCHFV NP-specific IgG in sheep from endemic CCHFV areas with high specificity, providing new insight into the heterogeneity of the immune response induced by natural infection with CCHFV in domestic animals.

## Introduction

Crimean-Congo hemorrhagic fever (CCHF) is a tick-borne disease affecting humans and in severe cases results in extensive hemorrhages and death. The disease is widespread in Africa, Asia, and Europe, where outbreaks of different magnitudes have occurred since CCHF was first recorded in 1944 (1). The World Health Organization considers CCHF a priority emerging disease due to its potential to cause severe epidemics without available countermeasures (2). Therefore, there is a need for the improvement of disease diagnostics and treatments, as well as the development of prophylactic vaccines.

The aetiological agent is the CCHF virus (CCHFV), a member of the genus *Orthonairovirus* of the order *Bunyavirales* (3). CCHFV possesses three negative-sense RNA segments: the S segment encoding the viral nucleoprotein (NP), the M segment encoding the envelope viral glycoproteins Gn and Gc, and the L segment encoding the viral polymerase (4). CCHFV is maintained in the environment by tick-vertebrate-tick cycles involving wild and domestic animals, such as rodents, birds, and ruminants (5, 6). Most common human transmission routes involve tick bites (tick to humans), as well as livestock handling (animal to humans, e.g., slaughtering) (7). Controlling the circulation and transmission of CCHFV within livestock populations using vaccination may be a valuable control measure in addition to human immunization (8). There are currently no CCHFV vaccines licensed for widespread use in humans and no available vaccines that prevent CCHFV infection in domestic animals.

Domestic animals infected with CCHFV do not show clinical signs of the disease and viremia only lasts between 2 and 9 days (9). As a result, serological detection of anti-CCHFV-specific antibodies as an indicator of the exposure of animals to the virus has been widely used to report CCHF seroprevalence among domestic animals in areas of investigation (6). Various studies have used modified protocols developed for testing human samples using whole CCHFV antigen or different in-house protocols using a single antigenic target, such as recombinant CCHFV antigen, NP (10–16). Currently, there is one commercial kit available for animal testing that also uses CCHFV NP as a single antigenic target, ID Screen^®^ CCHF Double Antigen Multi-species (17), while there are no assays that are specific for another CCHFV antigen. A good alternative antigen would be envelope glycoprotein CCHFV Gc, as this protein is responsible for the binding of CCHFV to the cell surface receptors facilitating entry. Additionally, it has been shown that CCHFV Gc is a target for neutralizing antibodies (18, 19).

In this study, we have developed two separate in-house assays, anti-CCHFV Gc-specific and anti-CCHFV NP-specific ELISA using commercially available antigens. We demonstrate that these two ELISAs can detect sero-reactivity in animals from endemic CCHFV areas, with high specificity. We also show that testing for antibodies that are specific to more than one CCHFV antigen demonstrates the heterogeneity of the humoral immune response to CCHFV in naturally infected sheep.

## Materials and methods

### Field serum samples

Well-characterized positive reference sera for CCHF were not available for this study. Sera from experimentally infected animals are not routinely available, and naturally infected animals do not display clinical signs. Therefore, evaluation of the assay performances was carried out using a range of sheep serum from various endemic CCHFV regions, as different strains of CCHFV may circulate (20), first from northern Tanzania (*n* = 12) and then from Bulgaria (*n* = 1,200). A subset of samples from Bulgaria (*n* = 80) was also tested using the commercial VectorCrimean-CCHF IgG (Vector Vest)^®^ and CCHF Double Antigen Multi-species (Innovative diagnostics) ID Screen^®^. ID Screen^®^ CCHF Double Antigen. The number of samples to be tested were estimated considering a (predetermined) diagnostic specificity of 98.0% (95% C.I 94.7–100%), 5% precision, and 90% power (21). Multi-species kit was run following the manufacturer's instructions, while Vector-Best's kit procedure was adapted for livestock species as was done previously (13), with HRP-conjugated Donkey anti-sheep/goat IgG (Bio-Rad) as a detection antibody that was diluted 1:30,000 in Blocker™ Casein. Results obtained *via* ID Screen^®^ CCHF Double Antigen Multi-species kit were expressed as sample vs. positive control ratio percentage (S/P %), while the results obtained *via* VectoCrimean-CHF IgG kit were presented as a sample vs. calculated cut-off value ratio (P/N), as per the manufacturer's instructions. In addition, sheep sera from non-endemic CCHFV area (United Kingdom; *n* = 200) collected as a part of surveillance were tested to assess the performance of the assays in samples that are expected to be negative for anti-CCHFV antibodies.

### Anti-CCHFV Gc-specific and anti-CCHFV NP-specific in-house ELISAs

Nunc MaxiSorp 96-well plates (Fisher Scientific) were coated overnight at 4°C with CCHFV antigens Gc or NP, at a concentration of 1 μg/ml in PBS. CCHFV Gc protein and CCHFV NP protein (strain IbAr10200, Nigeria, 1996) were produced in HEK293 cells (The Native Antigen Company, REC31696 and REC31639, respectively). The C-terminal transmembrane domain and the intravirion tail of recombinant CCHFV Gc have been replaced by a 15-amino acid glycine-serine linker and the human IgG1 Fc region whereas recombinant CCHFV NP incorporates N-terminal His-tag.

The next day antigen solution was flicked and plates were blocked with 200 μl of Blocker™ Casein in PBS (Thermo Scientific) for 2 h at room temperature. Wells were washed four times in 0.05% PBS-Tween20 (PBS/T). Test serum was diluted at 1:125 in Blocker™ Casein and 50 μl was added to the wells in duplicate on both CCHFV Gc- and CCHFV NP-coated ELISA plates. To ensure minimal plate-to-plate variation, appropriate blank (no sample, Blocker™ Casein only), positive (highly reactive sheep serum from the field), and negative (commercially available sheep serum (S3772), Sigma) controls were added in duplicates to each plate. The sheep serum used as a positive control was from the endemic CCHFV area and was selected based on having an OD value higher than the average OD value obtained for the 200 sheep sera from a non-endemic CCHFV area on both CCHFV Gc and CCHFV NP ELISAs. The plates were then sealed and incubated overnight at 4°C. The next day diluted sera were flicked and wells were washed four times in PBS/T. HRP-conjugated Donkey anti-sheep/goat IgG antibody (Bio-Rad) was diluted at 1:30,000 in Blocker™ Casein and 50 μl was added to each well and incubated for 1 h at room temperature. Plates were washed again in PBS/T four times. TMB substrate (Cambridge Bioscience) was added (50 μl/well) and incubated for 10 min at room temperature, protected from light before adding the stop solution (50 μl/well; Cambridge Bioscience) to conclude the assay. The optical density was read at a wavelength of 450 nm using a Vmax ELISA plate reader and Softmax Pro software (Molecular Devices).

### Immunogenicity study

To evaluate whether the proposed in-house ELISAs could detect antigen-specific responses, anti-CCHFV Gc-specific and anti-CCHFV NP-specific IgG levels were investigated in serum from sheep vaccinated with a Modified Vaccinia virus Ankara (MVA) vaccine candidate developed by Public Health England, and previously demonstrated to be immunogenic and protective in mice (22). MVA strain 1974/NIH clone 1 is a pox-vectored vaccine encoding the CCHFV glycoprotein (GP). Castrated lambs (*n* = 18), Charolais cross breed, aged 35–37 weeks were sourced for this study from a known supplier and were determined as clinically healthy based on examination by a qualified veterinarian before being entered into the study. All efforts were made to minimize animal suffering and endpoints were limited to a mild severity rating. Lambs were randomly allocated to one of the three study groups (*n* = 6) as follows: Group 1: 2 × 10^8^ plaque-forming units (pfu) of MVA-GP diluted in endotoxin-free phosphate-buffered saline (PBS)-prime only; Group 2: 6 × 10^7^ pfu per animal of MVA-GP diluted in endotoxin-free PBS-prime and boost; and Group 3: 2 × 10^8^ pfu of MVA-GP diluted in endotoxin-free PBS—prime and boost. Lambs were vaccinated on Day 0 (D0) intramuscularly, at the right base of the neck, using a low dead space luer lock (2 ml) syringe. Animals from groups 2 and 3 received a booster vaccination 28 days post vaccination (dpv). Lambs were monitored for adverse side effects by a qualified veterinarian. In addition, any health events were recorded throughout the study. Five milliliters of blood were collected from the jugular vein of each lamb on D0, 14, 21, 28, and 40 dpv after which animals were culled. All experimental procedures were conducted in accordance with the institutional animal use guidelines at the Animal and Plant Health Agency farm.

### Statistical analysis

Graphical representation and statistical tests of the data were performed in GraphPad Prism 8.4.3 or R 4.0.2 (23). The cut-off values of the in-house ELISAs were determined by applying a finite mixture model using the Expectation Maximization algorithm (24) where confidence intervals are computed by Monte Carlo simulations, suitable for large datasets. Calculations were performed in R using the R package “cutoff” (25). To estimate the diagnostic specificity, we used results from the 200 sheep sera samples from a non-endemic CCHFV area (“true negatives”). Diagnostic specificity was estimated by dividing the number of samples classified as negative, using the cut-off given by the finite mixture model, by the total number of true negatives. The confidence interval of the diagnostic specificity was also estimated (21). To assess the precision of the assays, the average coefficient of variation from plate to plate (inter-assay CV) using negative and positive control sample values and the average coefficient of variation between duplicates (intra-assay CV) were calculated and presented as mean and standard deviation (SD). Receiver operating characteristic (ROC) curve and area under the curve (AUC) were used to assess the performance of in-house ELISAs considering each commercial test, separately, as reference tests. Correlations were analyzed using Spearman's rank test where correlations above a threshold of r > 0.5 were considered significant. The Friedman test was used for multiple comparisons with Dunn's *post-hoc* analysis in sheep vaccinated with vaccine candidate. *P*-values < 0.05 were considered significant.

## Results

### Assessment of CCHFV Gc-specific and NP-specific sero-reactivity in domestic animals by in-house ELISAs

**Figure 1:**
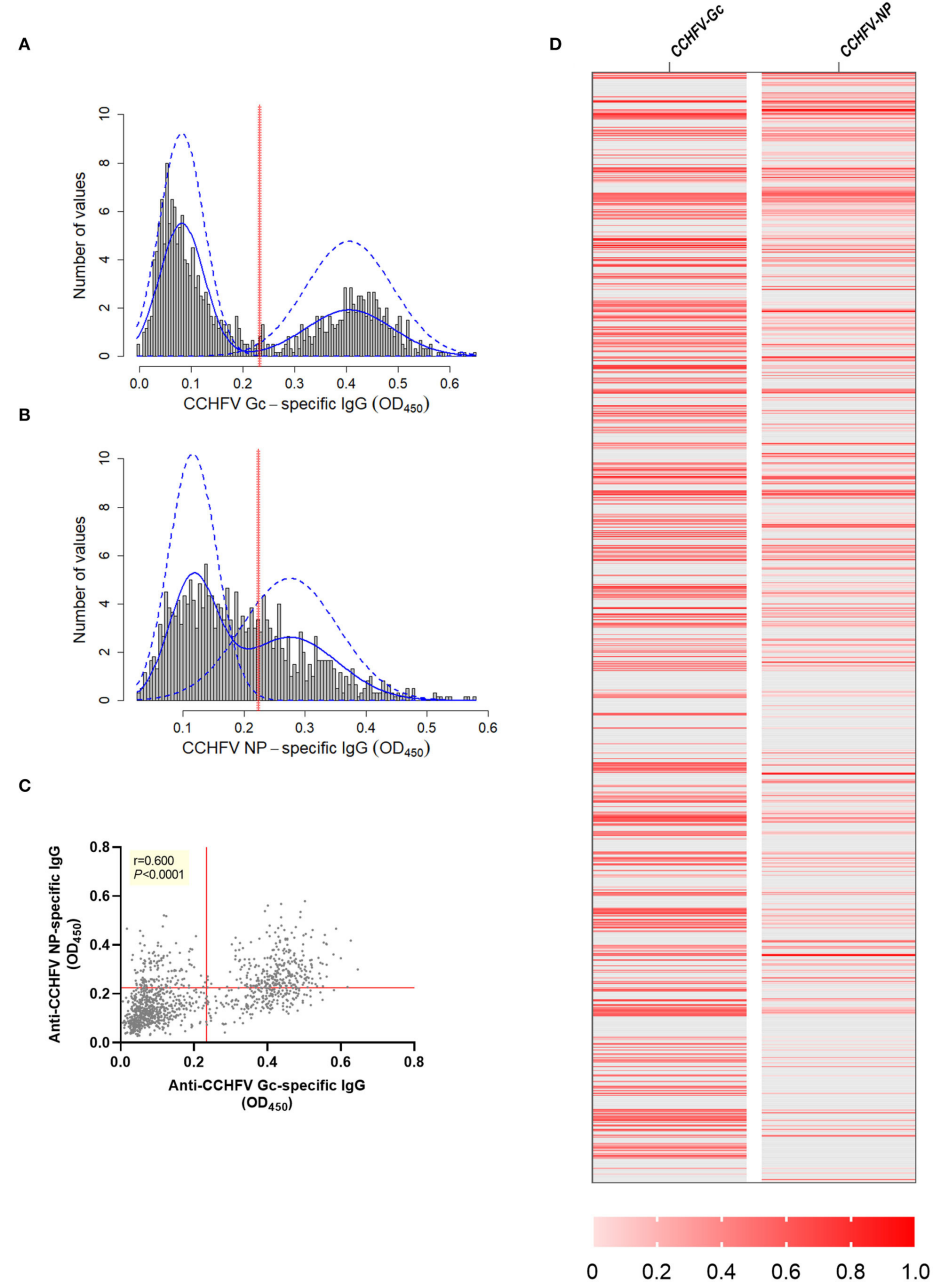
CCHFV Gc-specific and CCHFV NP-specific IgG sero-reactivity in sheep sera from endemic CCHFV areas. Sheep sera were collected as part of a cross-sectional study in an endemic CCHFV area (Bulgaria, *n* = 1,200) and tested for anti-CCHFV Gc-specific IgG levels **(A)** and anti-CCHFV NP-specific IgG levels **(B)** by in-house ELISAs. Data are represented as a histogram of the distribution of the OD_450_ values frequency (gray bars). Non-parametric estimation of the distribution (solid blue lines) and finite-mixture model (dashed blue lines) with estimated cut-off (red solid line). **(C)** Relationship between levels of anti-CCHFV Gc-specific and anti-CCHFV NP-specific IgG represented as correlation analysis (Spearman rank test) with red solid lines representing the estimated cut-off by finite mixed model. **(D)** Heatmap of data normalized across anti-CCHFV Gc and anti-CCHFV NP-specifc IgG using min-max normalization where minimum was defined as the cut-off specific for the assay as descriptive representation of the correlation at individual level. Each row represents data from one animal. Animals below the cut-off represented as gray and animals above the cut-off as different shades of red defined in the legend.

Anti-CCHFV Gc-specific and anti-CCHFV NP-specific in-house ELISAs were used for the determination of CCHFV antigen-specific IgG responses in sheep sera (n=1,200) sourced from endemic CCHFV areas in Bulgaria using a cross-sectional probabilistic study design. Frequency distributions of OD values for anti-CCHFV Gc-specific and anti-CCHFV NP-specific IgG were plotted (Figures 1A,B). Cut-offs estimated using a finite mixture model were 0.234 (0.232–0.236 95% C.I.) for anti-CCHFV Gc-specific-IgG and 0.225 (0.224–0.227 95% C.I.) for anti-CCHFV NP-specific IgG. After applying antigen-specific cut-offs, there were 38.417% of anti-CCHFV Gc-specific IgG sero-reactive animals above the cut-off (461/1,200) and 34.417 % of anti-CCHFV NP-specific IgG sero-reactive animals above the cut-off (413/1,200), while there were 23.333% (280/1,200) of animals that were sero-reactive to both CCHFV antigens. The 15.083% (181/1,200) of the animals were only sero-reactive to CCHFV Gc, while 11.083% (122/1,200) were sero-reactive only to CCHFV NP. And 50.5% (606/1,200) animals were below the cut-off for both antigens. Anti-CCHFV Gc-specific IgG levels showed a significant positive correlation with anti-CCHFV NP-specific IgG levels (Spearman *r* = 0.600, *P* < 0.0001) (Figure 1C). To allow visual comparison of individual animals across both assays, we generated a heat map that demonstrates the heterogeneity of the dataset (Figure 1D). When testing sheep sera from other CCHFV endemic areas (Tanzania, *n* = 12) with the same in-house ELISAs, results revealed similar anti-CCHFV Gc-specific and anti-CCHFV NP-specific sero-reactivity (Supplementary Figure 1).

Under controlled conditions, in sheep that were immunized using a prime/boost regimen with a candidate vaccine against CCHFV (CCHFV GP MVA vaccine), there was a modest increase in anti-CCHFV Gc-specific IgG, determined by anti-CCHFV Gc-specific in-house ELISA, 40 days post initial immunization in animals that received two doses of vaccine (group 2: 6 × 10^7^ pfu D0 vs. D40 *P* = 0.019 and D28 vs. D40 *P* = 0.035; group 3: 2 × 10^8^ pfu D0 vs. D40 *P* = 0.005 and D21 vs. D40 *P* = 0.035; Friedman test with Dunn's *post-hoc* analysis) (Supplementary Figure 2A). In addition, there was no increase in anti-CCHFV NP-specific IgG measured by the anti-CCHFV NP-specific in-house ELISA in all experimental groups, which was expected as the CCHF MVA vaccine encodes full-length CCHFV GP only (Supplementary Figure 2B).

### Diagnostic specificity and reproducibility of anti-CCHFV Gc-specific and anti-CCHFV NP-specific in-house assays

**Figure 2:**
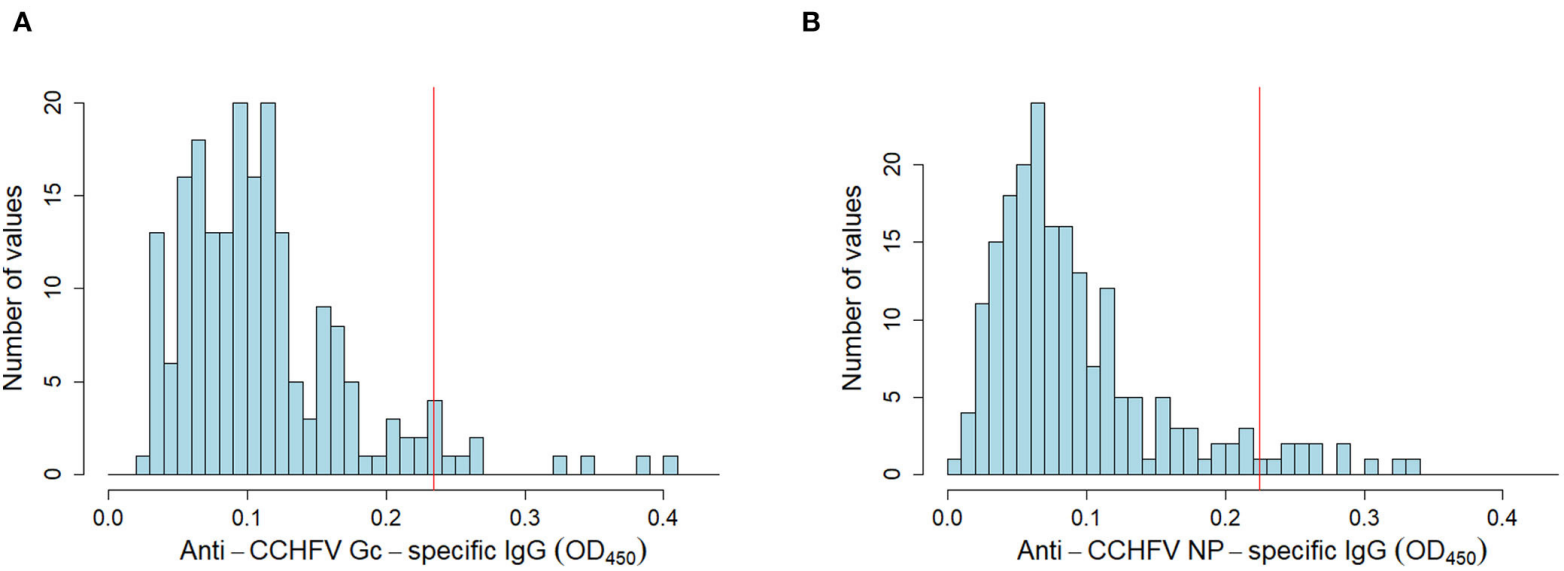
Anti-CCHFV Gc-specific and NP-specific IgG in sheep sera from non-endemic CCHFV areas. Sheep sera were collected from a non-endemic CCHFV country (*n* = 200) and tested for anti-CCHFV Gc-specific **(A)** NP-specific **(B)** IgG sero-reactivity by in-house assays. Data are represented as histograms of the distribution of the OD_450_ values frequency (blue bars). Estimated cut-off from finite-mixture model in an endemic area (same as Figure 1) is presented as a red solid line.

To estimate the diagnostic specificity of both assays, we tested sheep sera (*n* = 200) from a CCHFV non-endemic area, presented here as frequency distribution of OD values for anti-CCHFV Gc-specific and anti-CCHFV NP-specific IgG (Figures 2A,B). Diagnostic specificity for anti-CCHFV Gc-specific and anti-CCHFV NP-specific IgG in-house ELISAs estimated after the application of specific cut-offs evaluated by finite mixture model was 95.0% (95% C.I. 92.9–98.0%) for anti-CCHFV Gc-specific and 94.0% (95% C.I. 90.7–97.3%) for anti-CCHFV NP-specific ELISA.

Inter-assay coefficient of variation was 0.182 and 0.268 for CCHFV NP positive and negative controls, respectively, and 0.211 and 0.246 for CCHFV Gc positive and negative controls, respectively. The average intra-assay coefficient of variation was 0.051 for anti-CCHFV Gc-specific and 0.041 for anti-CCHFV NP-specific IgG ELISA demonstrating high precision of these assays (Supplementary Table 1).

### Comparative assessment of anti-CCHFV Gc-specific and NP-specific in-house assays and commercial VectoCrimean-CHF IgG and ID Screen^®^ CCHF double antigen multi-species kits

A subset of sheep sera from endemic CCHFV areas in Bulgaria (n = 80) tested with anti-CCHFV Gc-specific and anti-CCHFV NP-specific in-house ELISAs were also tested using commercially available VectoCrimean-CHF IgG and ID Screen^®^ CCHF Double Antigen Multi-species kits. VectoCrimean-CHF IgG commercial kit is intended for the detection of IgG to CCHFV antigen and we compared results following VectoCrimean-CHF IgG commercial kit with both in-house assays, anti-CCHFV Gc-specific and anti-CCHFV NP-specific IgG ELISAs. ID Screen^®^ CCHF Double Antigen Multi-species kit is a sandwich ELISA that is specific to a single antigenic target, CCHFV NP, which is also used as a detection agent in this assay (17). Therefore, a comparison between this assay and anti-CCHFV Gc-specific in-house ELISA was not performed.

**Figure 3:**
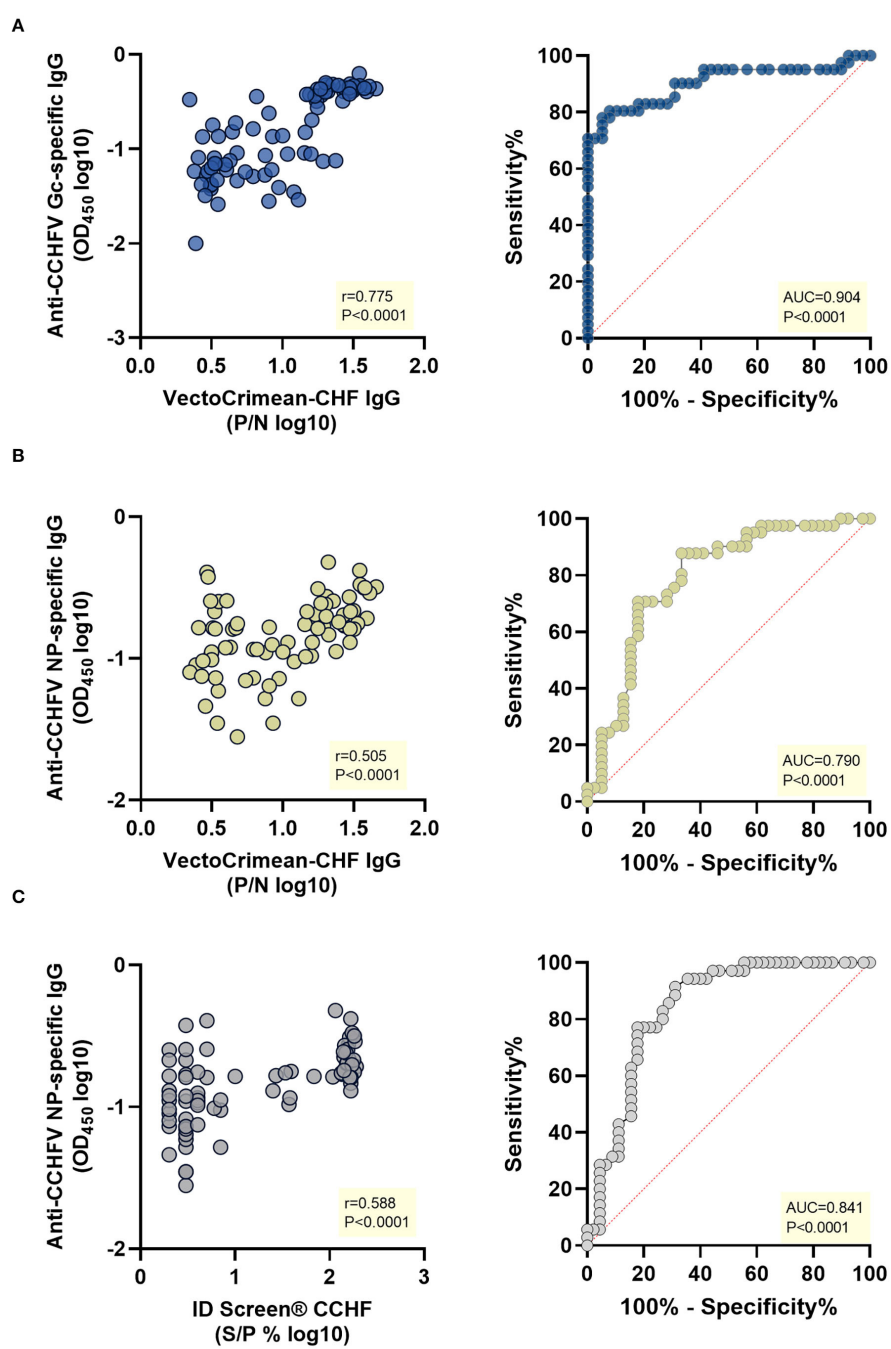
Correlation and receiver operating characteristic (ROC) analysis of anti-CCHFV Gc-specific and anti-CCHFV NP-specific IgG in-house ELISAs vs. VectoCrimean-CCHF IgG and ID Screen^®^ CCHF Double Antigen Multi-species commercial kits. A subset of sheep sera collected as part of a cross-sectional study in an endemic CCHFV area (*n* =8 0) was tested for anti-CCHFV Gc-specific IgG and anti-CCHFV NP-specific IgG by in-house ELISAs and with VectoCrimean-CHF IgG and ID Screen^®^ CCHF Double Antigen Multi-species commercial kits. Left panel shows spearman correlations between responses evaluated by: **(A)** anti-CCHFV Gc-specific IgG in-house ELISA and VectoCrimean-CHF IgG, **(B)** anti-CCHFV NP-specific IgG in-house ELISA and VectoCrimean-CHF IgG, or **(C)** ID Screen^®^ CCHF Double Antigen Multi-species. Right panel shows ROC curves generated using: **(A)** anti-CCHFV Gc-specific in-house ELISA OD measured at 450 nm and positive (*n* = 41) and negative (*n* = 39) sera results obtained with VectoCrimean-CHF IgG; **(B)** anti-CCHFV NP-specific in-house ELISA OD measured at 450 nm and positive (*n* = 41) and negative (*n* = 39) sera results obtained with VectoCrimean-CHF IgG, or **(C)** positive (*n* = 35) and negative (*n* = 45) sera results obtained with ID Screen^®^ CCHF Double Antigen Multi-species.

Using specific cut-offs according to the manufacturer's instructions of both commercial kits, samples were classified into positive and negative, and receiver operating characteristic (ROC) analysis was performed. The values for the area under the curves (AUC) were as follows: (i) 0.904 for anti-CCHFV Gc-specific in-house assay when using positive (*n* = 41) and negative (*n* = 39) samples evaluated by the VectoCrimean-CHF IgG assay (Figure 3A, right panel); (ii) 0.790 for anti-CCHFV NP-specific in-house assay when using positive (*n* = 41) and negative (*n* = 39) samples evaluated by the VectoCrimean-CHF IgG assay (Figure 3B, right panel) and (iii) 0.841 for anti-CCHFV NP-specific in-house assay when using positive (*n* = 35) and negative (*n* = 45) samples evaluated by the ID Screen^®^ CCHF Double Antigen Multi-species assay (Figure 3C, right panel).

When comparing theVectoCrimean-CHF kit results with anti-CCHFV Gc IgG in-house assay results, there were 32 samples that were positive on both assays (78.0%, Supplementary Figure 3A, left panel), while there were 36 samples tested negative on both assays (92.3%, Supplementary Figure 3A, right panel), resulting overall in a high positive correlation between two assays (Spearman *r* = 0.775, *P* < 0.0001, Figure 3A, left panel). When comparing the same VectoCrimean-CHF kit results to anti-CCHFV NP IgG in-house assay results, there were 13 samples that were positive on both assays (31.7%, Supplementary Figure 3B, left panel), while 34 samples tested negative on both assays (87.2%, Supplementary Figure 3B, right panel), resulting overall in moderate positive correlation between two assays (Spearman *r* = 0.505, *P* < 0.0001, Figure 3B, left panel). Comparative analysis of ID Screen^®^ CCHF Double Antigen Multi-species kit and anti-CCHFV NP IgG in-house assay revealed 13 samples that were positive (37.1%, Supplementary Figure 3B, left panel) and 40 samples tested negative on both assays (88.9%, Supplementary Figure 3B, right panel), resulting overall in a moderate positive correlation between two assays (Spearman *r* = 0.588, *P* < 0.0001, Figure 3C, left panel).

## Discussion

Domestic animals do not show clinical signs following infection with CCHFV but develop antibody responses against CCHFV (9). Serological assays have been used to indicate the presence of circulating CCHFV in areas where individuals might be at risk of exposure and infection. CCHFV NP has been considered highly immunogenic (26) and therefore used in the majority of serological assays as a detection antigen. Our study provides an assessment of two separate anti-CCHFV antigen-specific in-house assays, detecting IgG directed against CCHFV Gc and CCHFV NP. This is an important advance on previous serological tests available (commercial and in-house) that are specific to either one antigen (CCHFV NP) or whole CCHFV. Having assays that are detecting antibodies to different antigens would improve our understanding of the immunogenicity of CCHFV and the nature of antibody response in endemic settings.

In this study, we used anti-CCHFV Gc-specific and anti-CCHFV NP-specific IgG in-house ELISAs to determine the sero-reactivity toward CCHFV in endemic areas. In the absence of known positives and negatives from the affected area, we applied a finite mixture model to determine the cut-off. This method assumes that each dataset has a bimodal distribution, one representing a sero-positive and the other a sero-negative population. Identification of positive and negative populations within the results determines a cut-off as a best-fit value to discriminate between these two groups (24). This approach is more suitable to determine the cut-off when a serosurvey is performed in endemic areas and large number of samples are collected. A limitation of this method is that the cut-off might change when samples are collected in other endemic areas and therefore, should be estimated for each population.

Data presented here showed that both assays had high diagnostic specificity when considering non-vaccinated, non-infected animals from non-endemic CCHFV areas as true negatives; 95% for anti-CCHFV Gc-specific and 94% for the anti-CCHFV NP-specific IgG in-house ELISA. Anti-CCHFV NP-specific in-house ELISA diagnostic specificity is aligned with diagnostic specificity reported in other anti-CCHFV NP-specific assays (17, 27). However, assay protocols and methodologies to estimate diagnostic specificity differ across studies, including differences in species tested, study design, and sample size, and therefore they are not directly comparable. The same needs to be taken into account when comparing commercially available and in-house assays; relatively low accordance of anti-CCHFV NP IgG in-house ELISA positive results to both commercial kits used could be explained by differences in methodologies resulting in different assay performances. For example, the coating antigen concentration of CCHFV NP in ID Screen^®^ CCHF Double Antigen Multi-species kit is 5 ug/ml (17) while in anti-CCHFV NP in-house ELISA is 1 ug/ml.

It was not possible to estimate the assays' diagnostic sensitivity given that well-characterized positive reference sera in domestic animals were not available. In humans, the diagnostic sensitivity of the test can be evaluated against different stages of clinical disease (28). Previous studies in livestock and wild animals have used “positive” field samples as “true” positives; however, given that domestic animals do not show clinical signs, using field samples as true positives is questionable. Ideally, challenge studies should be conducted first to determine initial analytic and diagnostic sensitivity and specificity under controlled settings and complete the validation of the assays; however, as CCHFV is a BSL-4 pathogen, conducting studies like this in domestic animals is challenging. In this study, sheep immunized under controlled conditions using a prime/boost regimen with a candidate vaccine encoding full-length CCHFV GP only, showed modest anti-CCHFV Gc-specific IgG and absence of anti-CCHFV NP-specific IgG response, demonstrating our in-house assays have a good analytic specificity. The low post-immunization seroconversion observed may be the result of the MVA platform technology not inducing sufficient levels of immunogenicity in sheep, unlike if using other viral vectors for antigen delivery. For instance, it has been shown that immunization of mice with a single dose of a chimpanzee adenovirus (ChAd) against MERS-CoV can give humoral immunogenicity equivalent to two doses of MVA against MERS (29). Furthermore, it may be that in sheep, a heterologous prime-boost, with alternative viral vectors for both doses, maybe a preferable strategy to achieve increased seroconversion for more potent vaccine-induced humoral responses (30, 31).

In the sample sets from endemic CCHFV areas, we observed similar overall levels of sero-reactivity toward CCHFV Gc and CCHFV NP. However, these antibody responses show heterogeneity that might be caused by various factors, such as animal age, phase of the infection, time between exposure or different localities, and different decay rates of the antibody responses. The dynamic of the humoral immune response toward different CCHFV antigens in domestic animals is not known, specifically, whether animals develop anti-CCHFV NP and anti-CCHFV Gc specific antibodies at similar time points post-infection and how long both remain over time. In humans, data are suggesting that during different phases of CCHFV infection, there are IgG-specific responses directed against different CCHFV antigens, where IgG responses directed against CCHFV Gc were generated during acute infection (32), followed by IgG response toward CCHFV NP. Further analysis to formally assess potential differences in animals should be conducted. In addition, CCHFV shares antigenic similarities with other nairoviruses, such as the Nairobi sheep disease virus, Dugbe virus, Qalyub viruses, and Hazara virus (9, 33, 34). As a result, a cross-reaction might occur in places where these viruses are circulating simultaneously, or due to previous exposure, with important implications for seroprevalence estimations, surveillance programmes and vaccine development.

## Conclusion

The present study shows the development of two separate in-house assays, for the detection of anti-CCHFV Gc-specific and anti-CCHFV NP-specific IgG. To the best of our knowledge, this is the first report of an anti-CCHFV Gc-specific assay for the detection of IgG in domestic animal sera. Future studies using anti-CCHFV Gc-specific and anti-CCHFV NP-specific IgG in-house assays should reveal more details about the nature of antibody response following exposure to CCHFV to further support vaccine development, especially where differentiation between infected and vaccinated animals (DIVA) is preferable.

## Data availability statement

The data that support the findings of this study are available from the corresponding author upon reasonable request.

## Ethics statement

The sampling was conducted in accordance with the UK Home Office guidelines, Tanzania and Bulgaria. Different component of this study were approved by the Pirbright Institute and Animal Plant Health Agency (APHA) Biological Safety Committee via the Home Office, UK project licence PF10EF06F. Ethical approval for Tanzania was received from the Kilimanjaro Christian Medical University College, The Tanzania National Medical Research Ethics review committee NIMR/HQ/R.8a/Vol.IX/1069. Ethical approval for Bulgaria was received from the Bulgarian Food Safety Agency ethical committee (Approval number 178).

## Author contributions

SB-R, GL, BC, TL, and AL: conceptualization. SB-R, GL, EM, KH, EH, ST, TA, BM, BW, GB, NL, NB, KT, DW, JS, CB, and GW: data curation and writing. MC, RH, TL, and BC: secured funding. SB-R and GL: analysis of the data and methodology. SB-R, GL, and AL: writing original draft. TL and BC review and editing. All authors have read and agreed to the published version of the manuscript.

## Funding

This research was funded by the National Institute of Health Research (NIHR) [16/107/06] and the Biotechnology and Biological Sciences Research Council (BBSRC) [BB/R019991/1]. Tanzanian samples were collected as part of the Social, Economic and Environmental Drivers of Zoonotic disease (SEEDZ) study (grant no. BB/L018926/1, MTA NIMR/HQ/R.8a/Vol.IX/1069), from livestock in pastoral and agro-pastoral households in Arusha and Manyara districts.

## Conflict of interest

The authors declare that the research was conducted in the absence of any commercial or financial relationships that could be construed as a potential conflict of interest.

## Publisher's note

All claims expressed in this article are solely those of the authors and do not necessarily represent those of their affiliated organizations, or those of the publisher, the editors and the reviewers. Any product that may be evaluated in this article, or claim that may be made by its manufacturer, is not guaranteed or endorsed by the publisher.

## Supplementary material

Supplementary Figure 1 CCHFV Gc-specific and CCHFV NP-specific IgG sero-reactivity in sheep sera from endemic CCHFV areas. Sheep sera were collected from endemic CCHFV areas (Tanzania, *n* = 12) and tested for anti-CCHFV Gc specific IgG levels **(A)** and anti-CCHFV NP-specific IgG levels **(B)** by in-house ELISAs. Individual data points were expressed as OD measured at 450 nm and shown here as an aligned dot plot with bold horizontal lines showing the median. **(C)** Relationship between levels of anti-CCHFV Gc and anti-CCHFV NP-specific IgG represented as correlation analysis (Spearman rank test).

Supplementary Figure 2 Anti-CCHFV Gc-specific and anti-CCHFV NP-specific IgG response induced by CCHFV GP Modified Vaccinia virus Ankara (MVA) vaccine candidate. Sheep were primed (D0) with 2 × 10^8^ pfu (gray) or primed and boosted (D0/D28) with 6 × 10^7^ pfu (blue) or 2 × 10^8^ pfu (red) of CCHFV GP MVA vaccine candidate. Anti-CCHFV Gc-specific **(A)** and anti-CCHFV NP-specific **(B)** IgG response in sheep serum was quantified by in-house assays. Individual data points were expressed as OD measured at 450 nm and shown here as an aligned dot plot with bold lines showing the median with IQR. Dashed lines connect samples from the same animal. Assays were performed at D0, D14, D21, D28, and D40 post vaccination (*n* = 6 for all time points).

Supplementary Figure 3 VectoCrimean-CHF or ID Screen^®^ CCHF positive (right panel) or VectoCrimean-CHF or ID Screen^®^ CCHF negative (left panel) results were presented in relation to **(A)** anti-CCHFV Gc-specific IgG or **(B)** anti-CCHFV NP-specific IgG obtained by in-house ELISAs indicated either as gray (negative) or positive (red) based on the applied cut-off (dashed line). % in green indicate % of animals that are positive on both commercial kit and in-house ELISA, whereas the % in the yellow box indicates % of animals that are negative only on both assays.

Supplementary Table 1 Inter-assay and intra-assay coefficient of variation.
