# Supplementary material for: Development of anti-Crimean-Congo hemorrhagic fever virus Gc and NP-specific ELISA for detection of antibodies in domestic animal sera
Source: Front Vet Sci. 2022 Aug 25;9:913046. doi: 10.3389/fvets.2022.913046 (PMC9454098; doi:10.3389/fvets.2022.913046)
Supplement: Supplementary Figure 1 — CCHFV Gc-specific and CCHFV NP-specific IgG sero-reactivity in sheep sera from endemic CCHFV areas. Sheep sera were collected from endemic CCHFV areas (Tanzania, n = 12) and tested for anti-CCHFV Gc specific IgG levels (A) and anti-CCHFV NP-specific IgG levels (B) by in-house ELISAs. Individual data points were expressed as OD measured at 450 nm and shown here as an aligned dot plot with bold horizontal lines showing the median. (C) Relationship between levels of anti-CCHFV Gc and anti-CCHFV NP-specific IgG represented as correlation analysis (Spearman rank test). [file Data_Sheet_1.PDF]

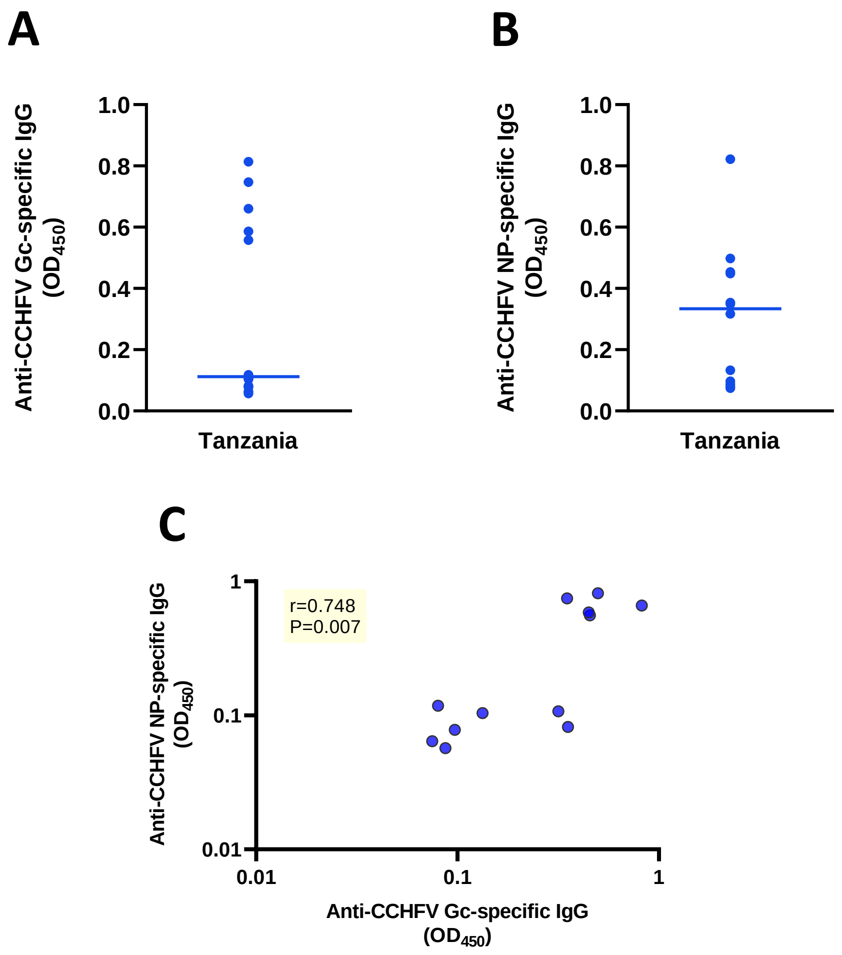
A
Anti-CCHFV Gc-specific IgG
(OD450)
1.0
0.8
0.6
0.4
0.2
0.0
Tanzania
B
Anti-CCHFV NP-specific IgG
(OD450)
1.0
0.8
0.6
0.4
0.2
0.0
Tanzania
C
Anti-CCHFV NP-specific IgG
(OD450)
1
0.1
0.01
r=0.748
P=0.007
0.01
0.1
1
Anti-CCHFV Gc-specific IgG
(OD450)

# Figure S1

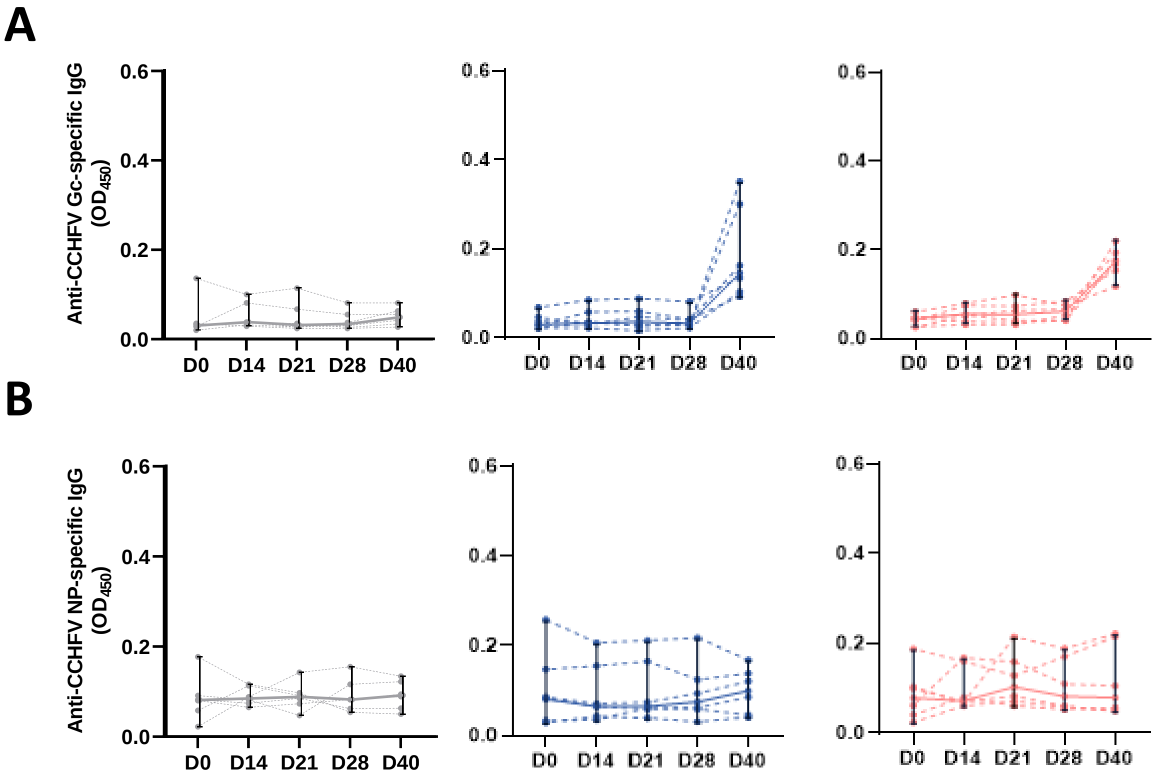
A
B
Anti-CCHFV Gc-specific IgG (OD450)
Anti-CCHFV NP-specific IgG (OD450)
0.0
0.2
0.4
0.6
D0
D14
D21
D28
D40

**Figure S2**

# Supplement Table 1

Inter-assay and intra-assay coefficient of variation

| | Inter-assay coefficient of variation (Mean; standard deviation) | | Intra-assay coefficient of variation |
|---|---|---|---|
| | *Controls* | | |
| **Anti-CCHFV Gc ELISA** | Positive | 0.212 | 0.051 |
| | Negative | 0.244 | |
| **Anti-CCHFV NP ELISA** | Positive | 0.184 | 0.041 |
| | Negative | 0.273 | |

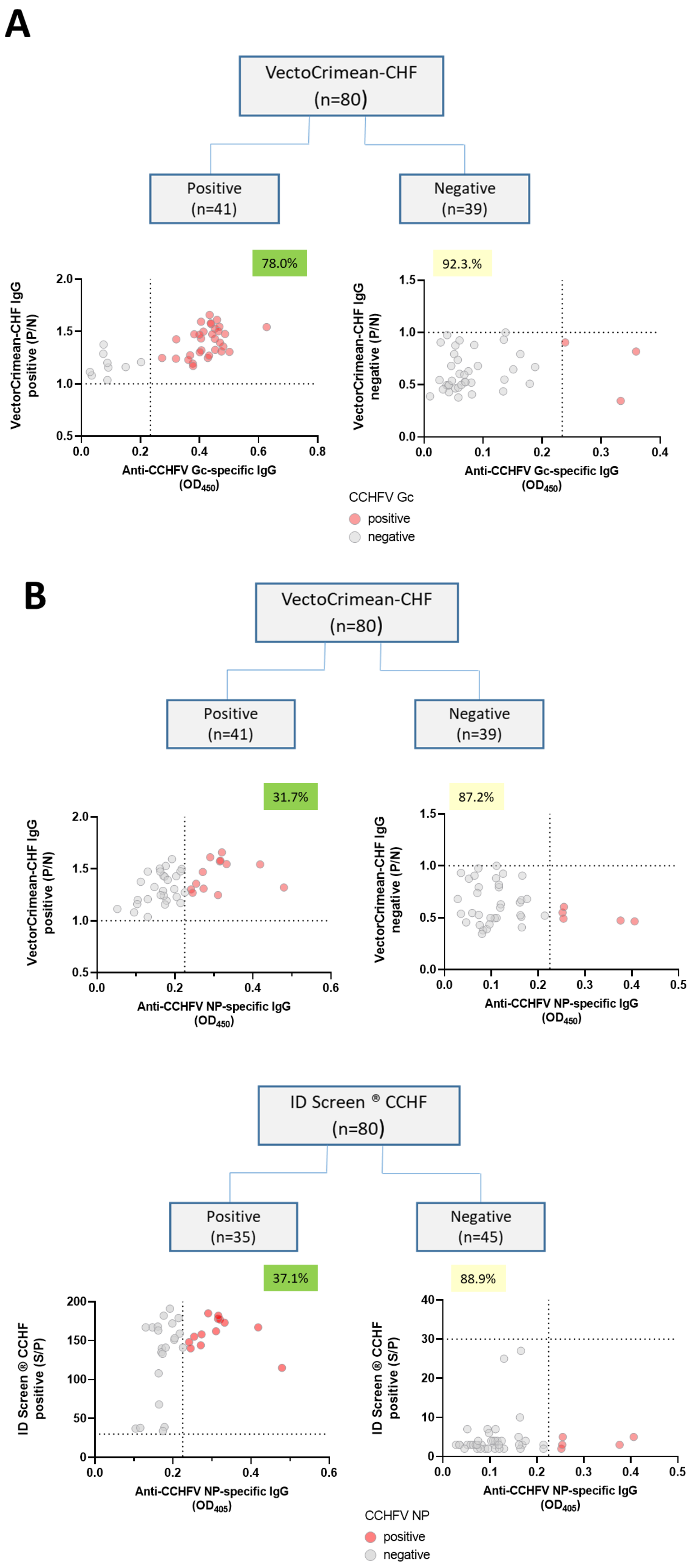
A
VectoCrimean-CHF
(n=80)
Positive
(n=41)
Negative
(n=39)
78.0%
92.3%
VectorCrimean-CHF IgG
positive (P/N)
VectorCrimean-CHF IgG
negative (P/N)
Anti-CCHFV Gc-specific IgG
(OD450)
CCHFV Gc
positive
negative
B
VectoCrimean-CHF
(n=80)
Positive
(n=41)
Negative
(n=39)
31.7%
87.2%
VectorCrimean-CHF IgG
positive (P/N)
VectorCrimean-CHF IgG
negative (P/N)
Anti-CCHFV NP-specific IgG
(OD450)
ID Screen ® CCHF
(n=80)
Positive
(n=35)
Negative
(n=45)
37.1%
88.9%
ID Screen ® CCHF
positive (S/P)
Anti-CCHFV NP-specific IgG
(OD405)
CCHFV NP
positive
negative

# Figure S3
